# Characterization of Magnesium Silicate Hydrate (MSH) Gel Formed by Reacting MgO and Silica Fume

**DOI:** 10.3390/ma11060909

**Published:** 2018-05-28

**Authors:** Tingting Zhang, Jing Zou, Baomin Wang, Zhenlin Wu, Yuan Jia, Christopher R. Cheeseman

**Affiliations:** 1 Faculty of Infrastructure Engineering, Dalian University of Technology, Dalian 116023, China; tingtingzhang@dlut.edu.cn (T.Z.); zou_jing8@mail.dlut.edu.cn (J.Z.); wangbm@dlut.edu.cn (B.W.); 2 School of Optoelectronic Engineering and Instrumentation Science, Dalian University of Technology, Dalian 116023, China; zhenlinwu@dlut.edu.cn; 3 Hebei Provincial Key Laboratory of Inorganic Nonmetallic, College of Materials Science and Engineering, North China University of Science and Technology, Tangshan 063210, China; 4 Department of Civil and Environmental Engineering, Imperial College London, London SW7 2AZ, UK; c.cheeseman@imperial.ac.uk

**Keywords:** hydration products, amorphous material, microstructure, MgO, silica fume

## Abstract

Magnesium silicate hydrate (MSH) gel was formed by reacting magnesium oxide (MgO) with silica fume (SF) in distilled water. The MSH was prepared using a MgO/SF molar ratio of 1.0 (40:60 weight ratio). Samples were analyzed during hydration process up to 300 days at room temperature. The MSH characterization has been carried out using a range of analytical techniques. Quantitative analysis was achieved using thermogravimetric analysis (TG/DTG) with a de-convolution technology. The structure of MSH gel was characterized using solid state nuclear magnetic resonance (^29^Si NMR) and Fourier transform infrared (FT-IR) spectroscopy. Transmission electron microscopy (TEM) was used to investigate MSH microstructure. Compared with natural magnesium silicate hydrate minerals, the structure of MSH gel is highly disordered and generates on the surface of particles, producing a shell structure with cavity. The molecular structure of MSH phase is layered. The results also show that the extent of polymerization of MSH gel is related to the solution pH during hydration.

## 1. Introduction

The MgO-SiO_2_-H_2_O system has been reported to have potential application for radioactive waste encapsulation [1,2,3,4,5,6,7]. Brew and Glasser have prepared magnesium silicate hydrate (MSH) gel by mixing Na_2_SiO_3_∙5H_2_O and Mg(NO_3_)_2_∙6H_2_O solutions at 20–25 °C and studied the alkali (K, Cs) sorption potential of the resultant synthetic gels [8,9]. The reduced pH of MSH formed by MgO-SiO_2_-H_2_O means this system has potential to be used for the encapsulation of mixed Aluminum and Mg(OH)_2_-rich (Magnox) wastes resulting from the nuclear industry [4,6]. The high alkaline pore solution in traditional radioactive waste repositories can result in a series of alterative reaction products and physicochemical changes over extended periods of time (such as the dissolution of clays) [10,11,12,13]. MSH cement with pH values 9.5–10.5 is within the range required for lower-pH (<11) cements to be considered to improve overall cement-clay compatibility [14,15]. In addition, good mechanical properties and thermodynamic stability of reaction produce in the MgO-SiO_2_-H_2_O system have been reported [16,17,18]. Due to the unique properties of the generated MSH gel, the understanding of the reaction mechanism and characterization of the microstructure and molecular structure have been studied recently [19,20,21,22,23,24,25,26,27,28,29,30,31,32,33,34,35,36,37,38,39].

MSH gel is the main hydration product of the MgO-SiO_2_-H_2_O system. Despite its amorphous nature and lack of long range order, its structure at the local scale can be expected to bear resemblance to crystalline magnesium silicates. There are two different crystal structure models for natural magnesium phyllosilicates, the serpentine-like model [21,22] (T-O layers; antigorite, chrysotile, lizardite [23,24,25,26,27,28]) and the talc-like model [26,27] (T-O-T layers; talc, sepiolite, palygorskite), where T indicates a sheet of interconnected Si–O tetrahedral and O stands for a sheet of Mg–O, which octahedrally coordinates to hydroxyl groups and to the apical oxygen atoms of the T sheet(s) [29]. 

^29^Si nuclear magnetic resonance spectroscopy (NMR) studies of these minerals have shown important molecular structure information. The serpentine-like model (T-O): ^29^Si NMR of chrysotile shows the major signal at −87.6 ppm, while the spectrum of serpentine exhibits one strong signal at −94.0 ppm due to difference in the interconnected Si-O tetrahedral angle in the molecular structure [30,31,32]. The talc-like model (T-O-T): the chemical shift for talc is −98 ppm and this represents the normal T-O-T layers found in magnesium silicates [8,33,34]. Distinguish from typical T-O-T layered mineral (talc), the major signals of sepiolite are at −92 ppm, −95 ppm and −98 ppm, respectively [23]. ^29^Si NMR exhibits resonance at −98 ppm due to the Q^3^ unit in the internal Si–O tetrahedral layer and resonance at −92 ppm due to the Q^3^ unit via inverted Si–O–Si linkages [29]. Inverted Si–O–Si linkages can cause open channels [34]. ^29^Si nuclear magnetic resonance spectroscopy (NMR) spectroscopy is most sensitive to the local ordering and structure around the spin nucleus and this permits structural studies not only of crystalline minerals but also of poorly crystalline silicate amorphous materials, such as MSH gel. The molecular structure analysis on MSH gel via ^29^Si NMR needs to depend on data of natural minerals.

MSH gel, precipitated by a mechanochemical process (Mg(OH)_2_, MgO and silicic acid), are determined by X-ray diffraction (XRD) to be poorly crystalline mixes of talc- and serpentine-like minerals, which is similar to the MSH gel produced hydrothermally by mixing Mg(NO_3_)_2_·6H_2_O and Na_2_SiO_3_·5H_2_O. There is also evidence that disordered MSH occurs naturally. Mitsuda [27] reports that MSH gel may be an intermediate product in the formation of talc. Solid-state ^29^Si NMR results indicate formation of a serpentine-like material at higher Mg contents, whereas lower Mg/Si gel produces more talc-like structures [8]. Fourier transform infrared spectroscopy (FT-IR) is also a useful tool to study amorphous gels and hydrated minerals. It is sensitive to vibrational modes, thereby indirectly provides information of the local atomic structure [35,36]. Natural magnesium silicate hydrate minerals show FT-IR absorption bands in four different regions, such as the Si–O vibration region (400–800 cm^−1^), Si–O stretching region (800–1300 cm^−1^), H_2_O and OH vibration region (1300–1800 cm^−1^) and H_2_O and OH stretching region (2800–4000 cm^−1^) [33,34,37,38,39]. The absorption bands of MSH gel can also reveal some unique properties of the material. 

In this work SF, an amorphous silica byproduct from silicon and ferrosilicon production, is used to react with light-burned MgO to form MSH gel. Samples are cured for up to 300 days at room temperature. The aim of the research is to understand the role of chemical reaction processes in MSH formation by quantitative analysis and the microstructure of the MSH phase. Hence MgO-SiO_2_-H_2_O samples have been prepared and the reaction products are analyzed with various characterization techniques. 

## 2. Materials and Methods

### 2.1. Sample Preparation

Light burned technical grade magnesium oxide with MgO activity index of 18 s was used in all experiments (Martin Marietta Magnesia Specialties, LLC., Manistee, MI, USA, MagChem^®^ Grade 30). SF was obtained from Elkem Materials Ltd., Shanghai, China (Elkem Microsilica^®^ Grade 955). The characterization data from the suppliers of the MgO and SF is given in Table 1. 

MgO and SF reacted in aqueous solutions at a 1:1 molar ratio (40:60 weight ratio). In order to aid full reaction, an excess water to solids (W/S) ratio of 10 was used, where S is the total mass of MgO and SF [40]. Samples were stored at room temperature (25 ± 1 °C) in 250 mL sealed polyethylene bottles for up to 300 days. The bottles were gently shaken throughout the hydration period to ensure that a homogenous solution concentration can be maintained for uniform reaction. The pH during hydration was measured at different curing stages using a pH meter (PHS-3C, Shanghai INESA and Scientific Instrument Company, Shanghai, China). The solid residue with different curing times was separated using filter paper with a pore size of 30–50 μm. The collected solids were soaked in absolute ethyl alcohol for 24 h to inhibit further hydration reactions and then dried at 40 °C for 48 h before characterization. 

### 2.2. Sample Characterization

The crystalline phases in hydrated solid residue samples were determined using XRD, (D8 Advance, Bruker, Karlsruhe, Germany, Max 2400 V diffractometer with Cu Kα radiation at a scan rate of 0.5° 2*θ* min^−1^). The residual MgO content in MgO/SF pastes was determined by quantitative X-ray diffraction (Q-XRD, Bruker, Karlsruhe, Germany, Cu Kα, 40 kV and 40 mA) using the K-value method, with TiO_2_ used as an internal standard material [41,42]. The ground MgO and internal standard material (TiO_2_) were uniformly mixed at a mass ratio of 1:1 in a mortar with ethanol. One non-overlapping X-ray diffraction peak of MgO and TiO_2_ was chosen. *I_MgO_* and *I_TiO_*_2_ are the integral intensity of the diffraction peak of MgO and TiO_2_ respectively. *K_MgO_* is defined as follows:

*K_MgO_* = *I_MgO_/I_TiO_*_2_ (1)

The mixed powders with a mass ratio of 20:80 (TiO_2_:Sample), as control specimens, were homogeneously mixed. The mass percentage of MgO in the mixture was calculated by Equation (2).

*w_MgO_* = (*I’_MgO_*/*I’_TiO_*_2_)(1/*K_MgO_*)(*w_TiO_*_2_/(100 *− w_TiO_*_2_)) × 100 (%), *w_TiO_*_2_ = 20 (%) (2)

where *w_MgO_* is the mass percentage of MgO in MSH system, *I’_MgO_* and *I’_TiO_*_2_ are equal to the integral intensity of the diffraction peak of MgO and that of TiO_2_ in the specimens.

The mass loss of MSH samples was determined using Thermogravimetric analysis (TG/DTG, Mettler Toledo, Zurich, Switzerland). The samples were hold in alumina crucibles and heated in a nitrogen atmosphere from room temperature to 1000 °C at 10 °C min^−1^ heating rate.

The local atomic structure in sample was determined using solid-state high-resolution 29Si NMR (Bruker Advance III 500 MHz spectrometer, Karlsruhe, Germany, field strength 9.4 T, operating frequency 79.5 MHz for 29Si). Samples for analysis were packed into 4 mm zirconia rotors and spun at 8 kHz. The 29Si spectra was acquired over 20,000 scans using a pulse recycle delay of 2 s, a 30° excitation pulse with 2 μs width, a relaxation delay of 60 s and an acquisition time of 0.0426 s. Various atomic nuclei can be quantitatively analyzed as the NMR spectrum area is in proportion to the number of spin nuclei in the same chemical environment. The total range of ^29^Si chemical shifts in the silicates was considerable, from −60 to −120 ppm, with analytically significant sub-division into well-separated ranges for monosilicates (Q^0^), disilicates and chain end groups (Q^1^), as well as middle groups in chains (Q^2^), chain branching sites (Q^3^) and the three-dimensional cross-linked framework (Q^4^) [43]. ^29^Si NMR spectra were superimposed by a few resonant peaks [44,45]. De-convolution was applied to assign resonances to individual species using PeakFitv4.12 (Peak separation and analysis software, SeaSolve Software Inc., San Jose, CA, USA).

The vibrational modes of MSH gel were determined using FT-IR. The infrared spectra were recorded using an IR spectrometer (EQUINOX 55, Bruker, Karlsruhe, Germany) with the samples suspended in KBr discs and pressed at 1.3 MPa. The spectroscopic work was conducted to analyze the broad IR absorption, appearing in the 400–4000 cm^−1^ region. 

The morphological structure of MSH gel was analyzed by transmission electron microscopy (TEM, Tecnai G2 F20/200 kV, FEI NanoPorts, Hillsboro, OR, USA). MSH samples were dispersed in alcohol and maintained as dilute suspension. Then the dilute suspension was dropped onto a copper grid and dried before TEM observation.

## 3. Results

### 3.1. XRD Analysis

**Figure 1:**
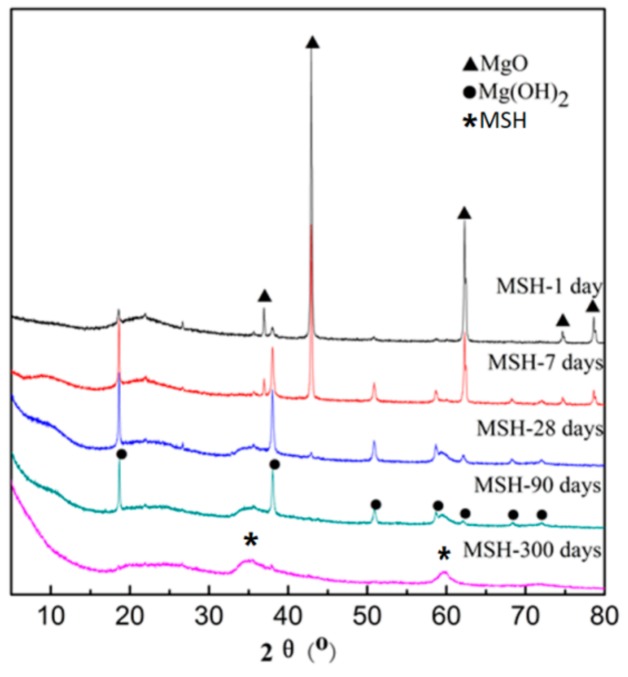
X-Ray diffraction (XRD) results of magnesium silicate hydrate (MSH) samples after curing for 1, 7, 28, 90 and 300 days.

Figure 1 shows the phase transformations occurring in the MgO-SiO_2_-H_2_O system with different curing times up to 300 days at room temperature. The two amorphous peaks at 2θ (35°) and 2θ (60°) are associated with the formation of MSH. After 7 days, MgO remains as the major crystalline phase but by 28 days the MgO has fully reacted to form Mg(OH)_2_ and amorphous MSH. The consumption of Mg(OH)_2_ is slow and residual Mg(OH)_2_ can be observed at 90 days. By 300 days all the Mg(OH)_2_ has been replaced by amorphous MSH. The main phases in MgO-SiO_2_-H_2_O system include MgO, Mg(OH)_2_, Silica fume, MSH gel and water. 

Based on Q-XRD method (2.2), *w_MgO_* (*n* days) can be calculated. The consumption percentage of MgO (*α_MgO_*) is calculated by Equation (3).

*α_MgO_* = (*w_MgO_* (0 day) − *w_MgO_* (*n* days)/*w_MgO_* (0 day)) × 100 (%) (3)

where *α_MgO_* is consumption percentage of MgO. *w_MgO_* (*n* days) represents residual MgO mass percentage (*n* is 1, 7, 28, 90 and 300) in MSH system and *w_MgO_* (0 day) represents initial MgO mass percentage in MSH system. The results are shown in Table 2.

### 3.2. TG/DTG Analysis

**Figure 2:**
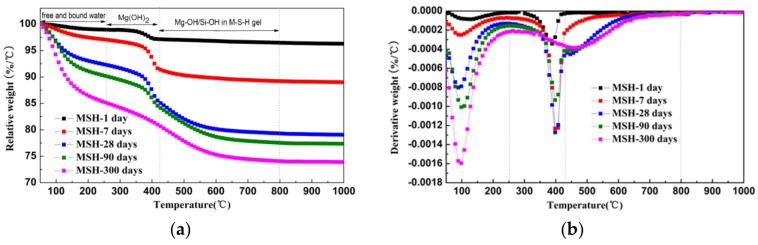
(**a**) Thermogravimetric (TG) and (**b**) Derivative weight curves (DTG) data for MSH samples cured for times up to 300 days.

**Figure 3:**
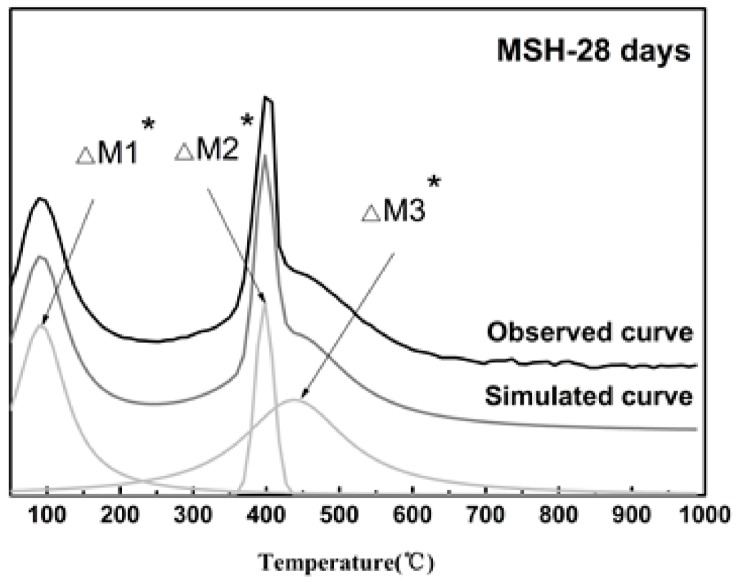
De-convolution of the DTG curves of MSH 28 day sample. The black line is the experimental data. The dark gray line is the global fitting curves and the light gray lines represent the Gaussian peaks on the x-axis obtained by the fitting procedure.

The corresponding TG/DTG data shows the relative weight loss and derivative weight curves of MSH samples at various ages, see Figure 2a,b. The reaction product is mixed with crystalline and amorphous phases. Attributing to overlapped weight loss peaks, the calculated value is lower than the actual value on the mass loss of MSH phase via traditional thermo-gravimetric analysis method. Based on the derivative thermo-gravimetric (TG/DTG) data, the integration area of derivative curves is equivalent to the weight loss. Therefore, the curve-fitting method based on the de-convolution technology can be used to analyze the DTG curves in order to obtain more precise calculation of each phase. Figure 3 demonstrates the analysis of MSH-28 days using curve-fitting method. Table 3 lists each Gaussian peak data of all the testing samples.

There are three weight loss stages that can be seen in Figure 2a. The first stage represents the weight loss of free and bound water (∆M1*) from room temperature to 250 °C. The free and bound water in reaction production increases with curing time. The water loss is related to the mass of MSH gels indirectly. According to the derivative weight curves (DTG) of MSH samples, the second mass loss can be observed in the 250–430 °C range, attributing to the de-hydroxylation of Mg(OH)_2_ (∆M2*). When heating temperature is over 430 °C, Mg–OH and Si–OH in MSH gels continue to remove the hydroxyl group. ∆M3* demonstrates the last weight loss stage and is consistent with the formation of MSH gel. The MSH gel content is normally obtained using the third thermo-gravimetric data.

The hydroxyl group content of MSH gel and Mg(OH)_2_ is confirmed by traditional thermo-gravimetric and curve-fitting method. Compared with DTG result, de-convolution data is more precise. *W_Mg_*_(*OH*)2_ (the mass of Mg(OH)_2_) and *W_–OH_* (the mass of hydroxyl groups of MSH gel) in the mixture are calculated by Equation (4) and Equation (5):

*W_Mg_*_(*OH*)2_ = (**∆**M2*/(100 − **∆**M1* − **∆**M2* − **∆**M3*)) × (*W_SF_* + *W_MgO_*) × *M*_{*Mg*(*OH*)2}_/*M*_{*H*2*O*}_ (4)

*W_–OH_* = (**∆**M3*/(100 − **∆**M1* − **∆**M2* − **∆**M3*)) × (*W_SF_* + *W_MgO_*) (5)

where: *W_Mg_*_(*OH*)2_ and *W_–OH_* are the mass of Mg(OH)_2_ (g) and the hydroxyl group of MSH gel (g). *M*_{*Mg*(*OH*)2}_ and *M*_{*H*2*O*}_ are molar mass of Mg(OH)_2_ (g/mol) and H_2_O (g/mol). *W_SF_* and *W_MgO_* are initial mass of SF (g) and MgO (g), respectively. 

### 3.3. ^29^Si MAS NMR Spectra

**Figure 4:**
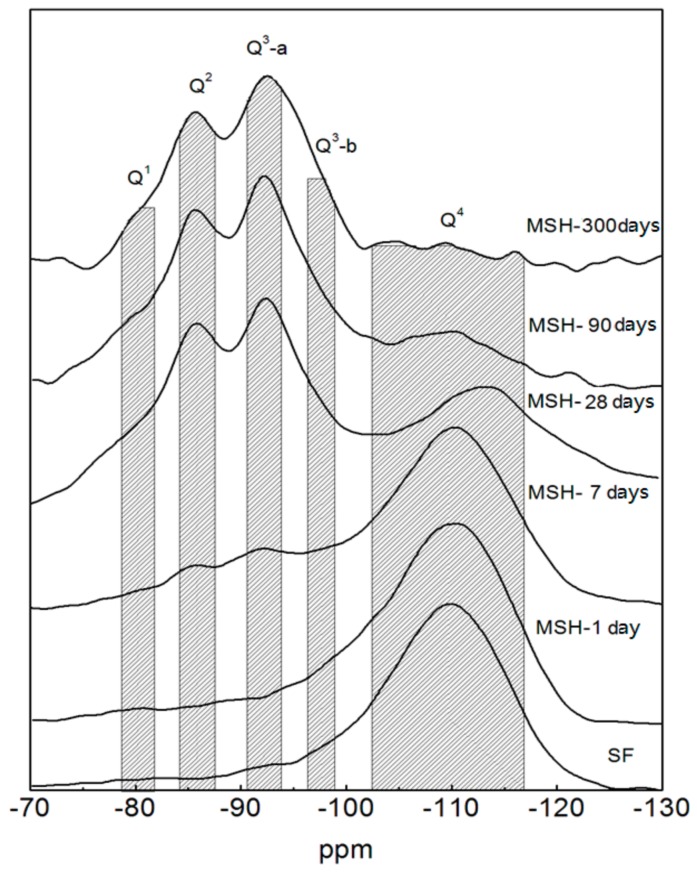
^29^Si MAS NMR spectra of the MSH samples for different curing time.

**Figure 5:**
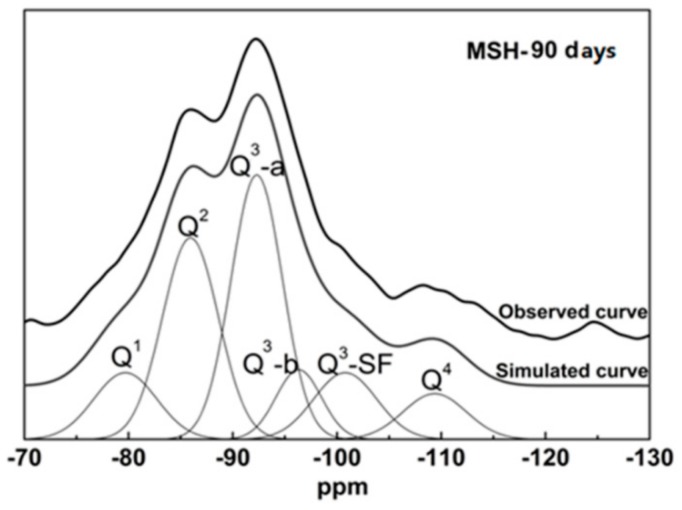
^29^Si MAS NMR spectrum of the MSH-90 days sample and the optimum de-convolution result.

**Figure 6:**
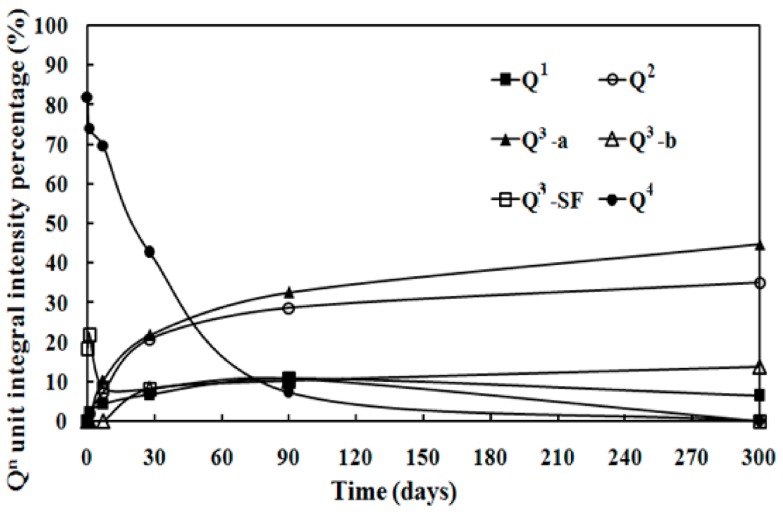
Q^n^ unit integral intensity percentage of the MSH samples via ^29^Si MAS NMR spectra for different curing time.

The ^29^Si MAS NMR spectra of the SF sample show a peak at around −110 ppm, which is assigned to the Q^4^ unit, see Figure 4. This is consistent with a cross-linked framework formed from silicon-oxygen tetrahedron. Figure 4 also shows the spectra of MSH samples produced at different curing times. The peaks of MSH gel, found at −80, −85, −92, −97 and −110 ppm respectively, reveal the decomposition of SF and the formation of MSH gel in the MgO-SiO_2_-H_2_O system [46]. According to the literature, the features at −97, −92, −85 and −80 ppm correspond to Q^3^-b, Q^3^-a, Q^2^ and Q^1^, respectively [7,29]. In Figure 4, Q^3^ at −97 ppm disappears after 300 days curing. Chemical shifts located around −100 and −110 ppm are consistent with Q^3^-SF and Q^4^ environment (unreacted SF residue). Q^3^-a is assigned to the Si unit via inverted Si–O–Si linkages [29], while Q^3^-b likely reflects the Si unit in the Si–O tetrahedral layer. The ^29^Si MAS NMR spectra were de-convolved into different fitted curves based on the ^29^Si site data. Figure 5 illustrates the analysis of MSH-90 days using curve-fitting method. The data and calculation results from the de-convolution of ^29^Si NMR spectra are given in Table 4. The variation tendency of different Si–O tetrahedral environment is shown Figure 6.

The sum of Q^3^-SF and Q^4^ represents residual SF. *I(Q^1^)*, *I(Q^2^)*, *I(Q*^3^) and *I(Q^4^)* are the integral intensity percentage (%) of the signals Q^1^, Q^2^, Q^3^ and Q^4^, respectively. *α_SF_* (the consumption percentage of SF) is calculated according to Equation (6):

*α_SF_* = 100 − (*I*(*Q*^4^) + *I*(*Q*^3^*-SF*)) (%) (6)

where *α_SF_* is consumption percentage of SF. Figure 6 indicates that the integral intensity of Q^4^ can be reduced significantly, while that of Q^2^ or Q^3^-b increases as the curing time prolongs. The integral intensity of silicon atom with other coordination shows little change.

Meanwhile, the average condensation degree (*CD*) can be worked out based on ^29^Si MAS NMR data. The average *CD* reflects the formation process and molecular structure changing tendency of MSH gel. The average *CD* of samples can be calculated using Equation (7):

*CD* = (3*I*(*Q*^3^-*b*) + 3*I*(*Q*^3^-*a*) + 2*I*(*Q*^2^) + *I*(*Q*^1^))/3(*I*(*Q*^3^-*b*) + *I*(*Q*^3^-*a*) + *I*(*Q*^2^) + *I*(*Q*^1^)) (7)

**Figure 7:**
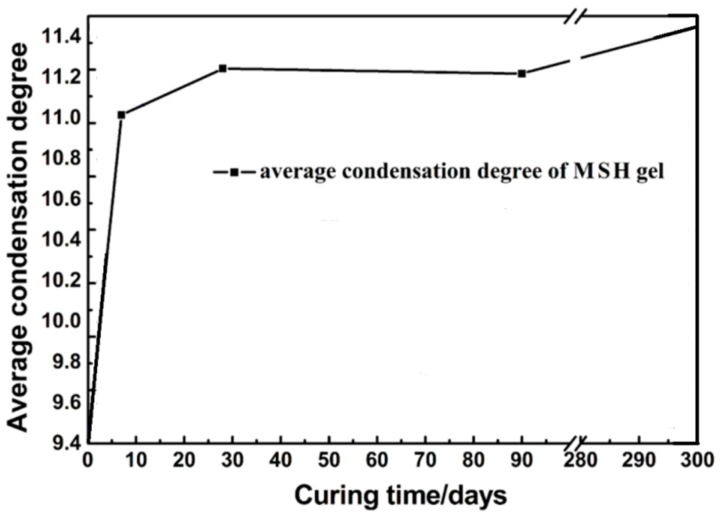
pH value and average condensation degree of cured MSH gel samples.

The *CD* results of MSH gels are shown in Figure 7. The average condensation degree rises quickly before 7 days and starts to level off (~0.8). After 90 days, the value slowly goes up to 0.84.

### 3.4. FT-IR Spectra

**Figure 8:**
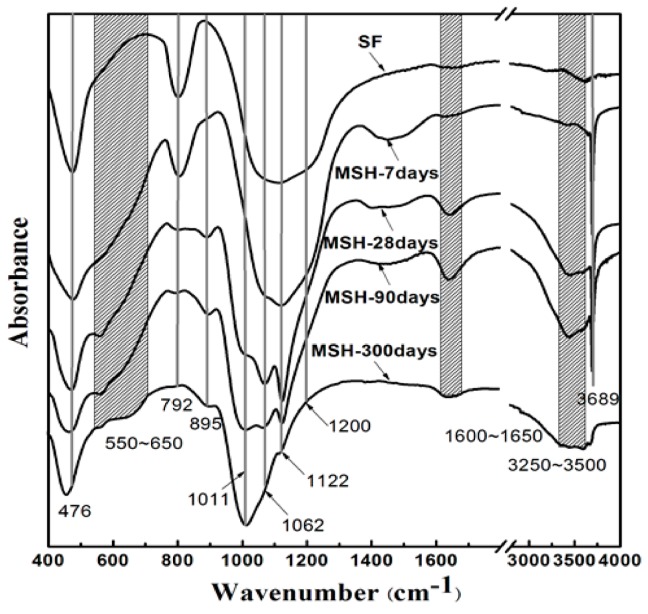
Fourier transform-infrared (FT-IR) spectra of the MSH samples and silica fume.

The FT-IR spectra of the MSH samples show changes in the hydration process of magnesium silicate hydrate cementitious material, indicating the reactions between MgO and SiO_2_, as shown in Figure 8. The SF (SiO_2_) shows absorption bands at 1000~1200, 792 and 476 cm^−1^, typical of four-coordinated silica, which can be assigned to asymmetrical stretching vibration, symmetrical stretching vibration and Si–O bending vibration, respectively [35,36]. The results also confirm that SF belongs to the framework structure. 

As the hydration process progresses, SF dissolves gradually as the silicon hydroxyl content increases. Figure 8 shows that the band at 1200 cm^−1^ shifts toward 1010 cm^−1^/1065 cm^−1^ (anti-symmetric stretching vibration of Si–O–Mg) and the 800 cm^−1^ band almost disappears [39,41,47]. This suggests that the framework silica atoms change into layer-structured magnesium silicate hydrate. A series of new bands appear from 550 to 650 cm^−1^ which can be attributed to the symmetric stretching vibration of Si–O–Mg of layer-structured magnesium silicate hydrate [33,34]. The band at 3690 cm^−1^ comes from the –OH stretching band of Mg(OH)_2_ and the weak band at 895 cm^−1^ comes from the stretching vibration of silanol groups (Si–OH) [41].

### 3.5. Transmission Electron Microscopy (TEM)

**Figure 9:**
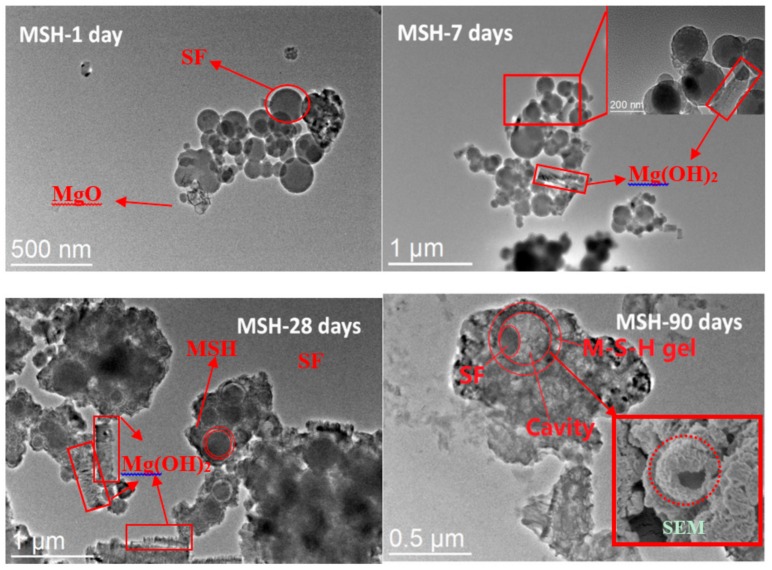
Transmission electron microscopy (TEM) bright field images of samples with different curing times. After 1 day, the spherical particles of the silica fume (SF) can still be found. As curing time extends, the particles gets covered and slowly consumed by MSH gel, forming the shell structure with cavity.

The micro-structure of reaction products of MgO and SF is shown in TEM images, see Figure 9. Initially, it can be found that MgO grains and SF particles coexist in pore solution from 1 day observation. After 7 days, MgO is hydrated to form Mg(OH)_2_ (stick-like morphology) and SF particles are covered in amorphous phase (MSH gel). After 28 days, the amorphous phase (MSH gel) grows around SF particles and the size of SF particles decreases due to the dissolution reaction in alkaline solution. After 90 days, MSH gel forms on the surface of particles but does not grow in the gap between the gel shell and particles. As a result, SF particles eventually dissolve and MSH phases form the shell structure with cavity.

## 4. Discussion

MgO transforms into Mg(OH)_2_ and MSH gel and into MSH gel eventually. Meanwhile SF gradually transforms into MSH gel. The mass of water in unreacted MgO-SiO_2_-H_2_O system equals to the mass of chemical structure water (–OH) in MSH gel with 300 days curing.

**Figure 10:**
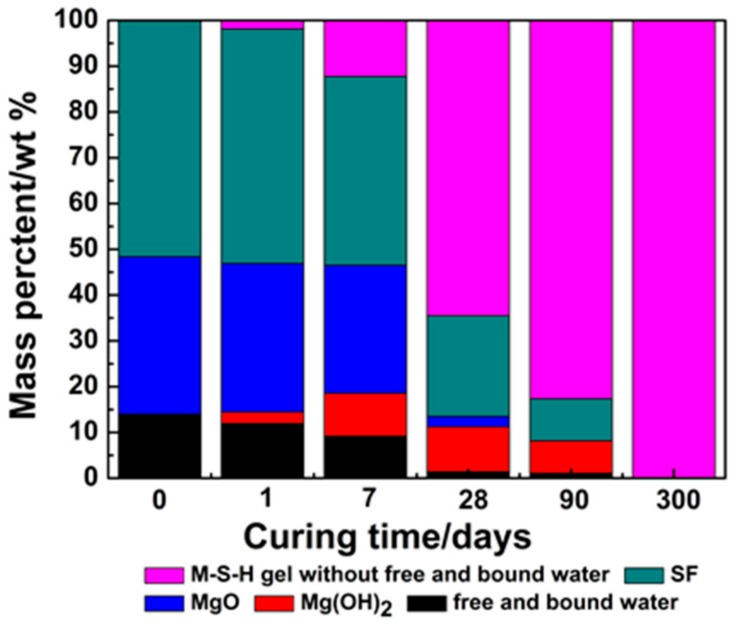
Quantitative analysis of the phase content of MSH samples cured for 300 days via multi-technique approach.

According to XRD, TG/DTG and ^29^Si NMR results, *W*_(*MSH gel*)_ (the mass content of MSH gel) and *W*_(*free and bound water*)_ (free and bound water) with different curing times can be calculated using Equations (8) and (9), as shown in Figure 10.

*W*_(*MSH gel*)_ = (*α_MgO_* × *W_MgO_* − *W*_*Mg*(*OH*)2_ × *M*_{*MgO*}/_*M*_{*Mg*(*OH*)2}_) + *α_SF_* × *W_SF_* + *W*_–*OH*_ (8)

*W*_(*free and bound water*)_ = *W*_–*OH*_(300 days) − (*M*_{*H2O*}_/*M*_{*Mg*(*OH*)2}_) × *W*_*Mg*(*OH*)2_(*n* days) − *W*_–*OH*_(*n* days) (9)

where *W*_(*MSH gel*)_ represents MSH gel formation mass (g) and *W*_(*free and bound water*)_ represents free and bound water mass with different curing times deducting free and bound water mass in MSH gel with 300 days curing. *α_MgO_* and *α_SF_* are consumption percentage of MgO and SF. *M*_{*MgO*}_ and *M*_{*Mg*(*OH*)2}_ are molar mass of MgO (g/mol) and Mg(OH)_2_ (g/mol). *W_Mg_*_(*OH*)2_ (*n* days) and *W*_–*O*_*_H_* (*n* days) are the mass of Mg(OH)_2_ (g) and the hydroxyl groups of MSH gel (g) with different curing times.

**Figure 11:**
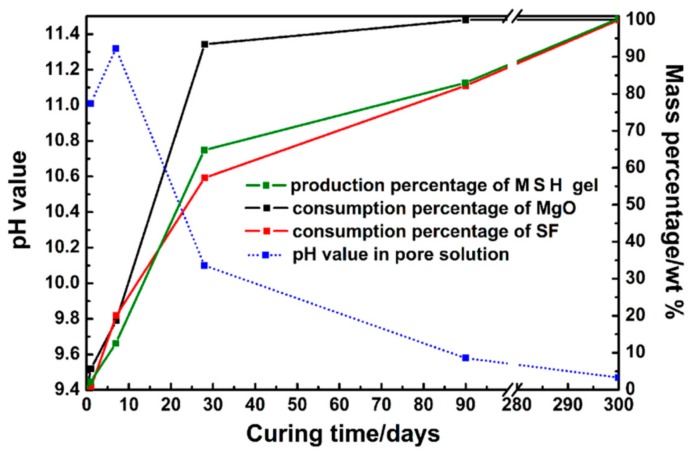
Consumption percentage of MgO/SF and production percentage of MSH gel in MSH samples cured for 300 days.

The formation of MSH gel is related to the hydrolysis of MgO and the dissolution of SF. According to quantitative data, phase transformations are shown in Figure 10 and Figure 11. The formation rate of MSH gel is determined by the hydration rate of MgO and the dissolution rate of SF. Li et al. investigate the performance of MgO-SiO_2_-H_2_O system based on the dissolution rate of MgO [41]. Figure 11 illustrates the consumption of MgO and SF and suggests MgO reaction rate is faster than SF dissolution rate, leading to the formation of magnesium hydroxide. Slow process plays a key role in a series of reactions. Therefore, MSH gel formation is determined by SF dissolution. 

The hydrolysis of MgO affects the pH value of the solution directly. The pH value is increased to about 11.3 after 7 days curing and then decreased to 9 after 90 days, see Figure 11. According to the NMR and FT-IR results, SF dissolves quickly during the first 28 days, however, due to the drop of pH level (pH ≈ 9), the dissolution rate is then decreases as the curing time grows. MSH gel formed on the surface of SF particles and then the SF particles are gradually consumed by diffusion through the MSH shell. According to Figure 9, MSH gel grows on the surface of particles and towards the solution but not fill the gap between gel shell and particle, contributing to shell structure formation.

From Figure 4, Figure 5 and Figure 6, it can be found that SF dissolves gradually in alkaline solution. Compared with hydrated magnesium silicate crystal (such as talc model or sepiolite model), the structure of MSH gel is more disordered. On the one hand, Si–O ribbons (Q^2^) can be formed and linked via Si unit correspond to Q^3^-b, which will result in the formation of hydrated magnesium silicate layers [33,34]. On the other hand, like sepiolite, hydrated magnesium silicate layers link to each other via inverted Si–O–Si, while open channels are formed. 

The Si-O-Si bond angle of the typical continuous layer silicates (Q^3^-b/−97 ppm) is about 120°. The resonance (Q^3^-a) found at −92.5 ppm indicates a decrease in Si–O–Si angles. The Q^3^ unit, via inverted Si–O–Si links, also increases the possibility to form open channels between ribbons [43]. According to TG/DTG data, the adsorbed water molecules in the channels of MSH gel increase as curing time grows in Figure 2 and Figure 8. These channels normally contain two types of adsorbed water molecules: one of them is coordinated to magnesium at the edge of octahedral strip and other type is the hydrogen-bonded to the silicate ribbons. The adsorbed water molecule is a key factor in Si–O–Si angle and chemical shift of MSH gel [23].

According to the trends of hydration products content transformation (Figure 1, Figure 8, Figure 9, Figure 10 and Figure 11) and the average condensation degree variation of MSH gel (Figure 7), hydration process of MgO-SiO_2_-H_2_O system can be divided into four stages: 

**The first period (0–10 days):** As soon as MgO gets into contact with water, there is a rapid consumption of MgO and pH value in solution increases to around 11.0. The reaction rate of SF and MSH gel is slow. MgOH^+^·OH^−^ formation occurs on the surface of MgO and high pH value inhibits further hydration. SF dissolution accelerates with time and gradually transforms nesosilicate and dimeric-tetrahedron. Meantime, MSH gel formation rate is limited by magnesium hydroxide precipitation and silicate concentration in solution [47,48,49]. MSH gel forms on the surface of SF particles.

**Thesecond period (10–30 days):** In this stage, the reduction in pH and the increase of magnesium ion and silicate concentration in solution promote the formation of MSH gel [47]. The consumption percentage of MgO and SF can reach maximum value and magnesium hydroxide transforms to MSH gel as well. MSH gel layer thickens and shell structure starts to form. The dissolution rate of SF determines the MSH gel generation. The Q^3^-a unit, as inverted Si-O tetrahedron, increases significantly and creates open channels between ribbons. 

**Thethird period (30–300 days):** MSH gel formation rate gradually increases as the SF dissolution rate grows. MSH gel grows on the surface of particles and has contact with the solution, however, it does not grow in the gap between the gel shell and particles, leading to the formation of the shell structure.

## 5. Conclusions

Hydration in the MgO-SiO_2_-H_2_O system can be divided into four stages: pre-induction period (0–1 days), the dormant period (1–10 days), the accelerating period (10–30 days) and the stable period (30–300 days). The pH value of the pore solution in the MgO-SiO_2_-H_2_O system is directly related to the hydration process. SF dissolution rate is slower than the MgO reaction rate, which has a key role in MSH gel formation. SF particles gradually dissolve and form a unique, shell structure with cavity. MSH gel forms on the surface of particles and toward the solution and grows on the surface to form a cavity in the gel shell, which is likely to cause shrinkage of the reaction products from the MgO-SiO_2_-H_2_O system. The extent of Si–O tetrahedron polymerization in MSH gels is related to the pH and ion concentration in the pore solution. There is a unique molecular structure in MSH gel. Si–O ribbons, which link with each other via inverted Si–O–Si, are formed during the first 7 days of reaction, which leads to the formation of hydrated magnesium silicate layers as the reaction progresses. The Q^3^ unit, via inverted Si–O–Si links, also enhances the possibility to generate open channels between ribbons while more water molecules are adsorbed in the channels.

## Figures and Tables

**Table 1:** Characteristics of the raw materials (manufacturer data).

Oxide (wt %)	MgO	SF
MgO	97.2	1.18
CaO	0.80	0.68
SiO_2_	0.35	93.71
Fe_2_O_3_	0.15	0.24
Al_2_O_3_	0.10	0.23
Na_2_O	-	0.35
K_2_O	-	1.74
SO_3_	0.02	0.37
P_2_O_5_	-	0.38
Cl	0.35	-
Loss in ignition	1.03	1.12
Bulk Density, loose (g/cm^3^)	0.35	0.28
Mean Particle Size (μm)	6.7	0.3
BET surface area (m^2^/g)	26	18

**Table 2:** Consumption percentage of MgO at curing time.

Curing time (day)	0	1	7	28	90	300
Consumption percentage of MgO (%)	0	7	18	92	100	100

**Table 3:** Weight loss data at three stages via traditional thermo-gravimetric analysis and using the curve-fitting method.

Sample ID	De-Convoluted Data (wt %)		
	∆M1*	∆M2*	∆M3*
MSH-1 day	2 ± 1	1 ± 1	2 ± 1
MSH-7 days	2 ± 1	4 ± 1	4 ± 1
MSH-28 days	8 ± 2	3 ± 1	11 ± 2
MSH-90 days	10 ± 2	2 ± 1	11 ± 2
MSH-300 days	15 ± 2	-	11 ± 2

**Table 4:** ^29^Si nuclear magnetic resonance (NMR) chemical shifts (ppm) and relative intensities (%) from de-convolution of the ^29^Si MAS NMR spectra for the MSH samples *.

Sample ID	Q^1^		Q^2^		Q^3^		Q^4^	
	Center (ppm)	Area (%)	Center (ppm)	Area (%)	Center (ppm)	Area (%)	Center (ppm)	Area (%)
SF	-	-	-	-	101.0 (sh/Q^3^-SF)	18 ± 3	−110.2 (p)	82 ± 6
MSH-1 day	−80.6 (p)	2 ± 1	−88.9 (p)	2 ± 1	−99.1 (sh/Q^3^-SF)	22 ± 3	−110.3 (p)	74 ± 5
MSH-7 days	−80.7 (p)	4 ± 1	−85.5 (p)	7 ± 1	−92.1 (p/Q^3^-a)	10 ± 1	−110.3 (p)	70 ± 5
					−98.9 (sh/Q^3^-SF)	8.5 ± 1		
MSH-28 days	−79.7 (p)	6.7 ± 1	−85.9 (p)	21 ± 2	−92.6 (p/Q^3^-a)	21.7 ± 3	−112.9 (p)	43 ± 3
					−97.2 (sh/Q^3^-b)	8.1 ± 1		
MSH-90 days	−79.7 (p)	11 ± 1	−85.9 (p)	29 ± 3	−92.3 (p/Q^3^-a)	33 ± 3	−109.4 (p)	7 ± 1
					−96.3 (sh/Q^3^-b)	10 ± 1		
					−100.8 (sh/Q^3^-SF)	11 ± 1		
MSH-300 days	−80.5 (p)	6 ± 1	−85.7 (p)	35 ± 3	−92.6 (p/Q^3^-a)	45 ± 3	-	-
					−97.2 (p/Q^3^-b)	14 ± 1		

* p—peak, sh—shoulder, Q^1^—Q^1^(3OH), Q^2^—Q^2^(2OH), Q^3^-a—Q^3^(OH) as continuous layer silicates, Q^3^-b—Q^3^(OH) as inverted silicates, Q^3^-SF—Q^3^(OH) in SF.
